# Hot plasmonic electrons for generation of enhanced photocurrent in gold-TiO_2_ nanocomposites

**DOI:** 10.1186/s11671-014-0710-5

**Published:** 2015-02-05

**Authors:** Lorcan J Brennan, Finn Purcell-Milton, Aurélien S Salmeron, Hui Zhang, Alexander O Govorov, Anatoly V Fedorov, Yurii K Gun’ko

**Affiliations:** School of Chemistry and CRANN Institute, Trinity College Dublin, Dublin 2, Ireland; Department of Physics and Astronomy, Ohio University, Athens, OH 45701 USA; ITMO University, 197101 Saint Petersburg, Russia

**Keywords:** Surface plasmon resonance, Hot electrons, Au-TiO_2_ nanocomposite, TiO_2_ films

## Abstract

In this manuscript, for the first time, we report a combination of electrophoretic and sintering approaches for introducing gold nanoparticles into nanoporous TiO_2_ films to generate ‘hot’ electrons resulting in a strong enhancement of photocurrent. The Au-TiO_2_ nanocomposite material was prepared by the electrophoretic deposition of gold nanoparticles into a porous nanoparticulate titanium dioxide film, creating a photoactive electrode. The composite film demonstrates a significant increase in the short circuit current (*I*_sc_) compared to unmodified TiO_2_ when excited at or close to the plasmon resonance of the gold nanoparticles. Then, we employed a thermal ripening process as a method of increasing the *I*_sc_ of these electrodes and also as a method of tuning the plasmon peak position, with a high degree of selectivity. Photo-electrochemical investigations revealed that the increase in photocurrent is attributed to the generation and separation of plasmonically generated hot electrons at the gold/TiO_2_ interface and also the inter-band generation of holes in gold nanoparticles by photons with *λ* < 520 nm. Theoretical modelling outputs perfectly match our results obtained from photo-physical studies of the processes leading to enhanced photocurrent.

**Electronic supplementary material:**

The online version of this article (doi:10.1186/s11671-014-0710-5) contains supplementary material, which is available to authorized users.

## Background

Surface plasmon resonance (SPR) is a feature of many metal nanostructures, based upon the collective oscillation of nanoparticle conduction band electrons, when excited at the particles plasmon resonance frequency [1]. SPR has been the subject of intense research in recent times due to the variety of potential important applications in areas such as imaging [2,3], photonics [4] and sensing [5-7]. SPR is a highly tuneable process and can be observed from the UV to the near-IR region of the electromagnetic spectrum, with plasmon resonance dependent upon the material used, the surrounding medium of the particles and the size and shape of the particular nanostructure. SPR has also attracted a great deal of attention as a plausible tool for increasing the efficiency of solar energy conversion devices. SPR has several potential applications in solar energy devices. SPR has been used to decrease the thickness of the absorber layer material in order to decrease bulk recombination currents, increase the optical path of incident light in the absorber layer and as efficient mechanism for light coupling into solar cells [8-16]. Recent research attention has focused on using plasmonic particles as the principle absorbers for the generation of photocurrent in solar energy devices. It has been demonstrated that excitation of a metal nanoparticles plasmon can cause a charge separation of electrons and holes at a metal semiconductor interface [17-23]. The resulting injection of ‘hot’ electrons into the semiconductor can generate a substantial photocurrent being observed at the plasmon wavelength. These observations infer that plasmonic particles can serve as a potential light-harvesting mechanism, enabling the generation of photocurrent. The advantage of using such a system lies in the fact that the plasmonic particles are highly tuneable across a wide range of wavelengths and are extremely stable and robust materials for solar energy harvesting. In addition, metal nanoparticles can absorb light much more efficiently when compared to semiconductors and dye molecules. Therefore, the use of photo-excited plasmonic electrons is potentially very attractive for applications in photochemistry and photo-catalysis [24-32], solar energy harvesting (solar cells) [8-12,20,33,34] and optoelectronics [23,35-39]. For example, in their work, Halas et al. have demonstrated that photons coupled into a metallic nano-antenna can excite resonant plasmons, which decay into energetic hot electrons injected over a potential barrier at the nanoantenna-semiconductor interface, producing a photocurrent [23]. This research opened up a range of potential applications including the use in on-chip silicon photonics, silicon light-harvesting devices, such as silicon-based solar cells, photodetectors and many other optoelectronic devices. In another work, the same group reported that a metallic nanoantenna can inject hot electrons into a nearby graphene structure, effectively doping the material [36]. The hot electron-doped graphene is a new type of hybrid material that is very promising for optoelectronic device applications such as optical switches, photodetectors and optically induced electronics. The same group has also demonstrated that nanoscale antennas can be sandwiched between two graphene monolayers yielding a photodetector with an 800% enhancement of the photocurrent relative to the analogous antenna-free graphene device. It was shown that the antenna contributes to the photocurrent in two ways: by the transfer of hot electrons generated in the antenna structure upon plasmon decay and by direct plasmon-enhanced excitation of intrinsic graphene electrons. This enables the device to achieve up to 20% internal quantum efficiency in the visible and near-infrared regions of the spectrum [35]. Hot electrons can also be used in photocatalysis. For example, it was reported that the H_2_ molecule can dissociate on gold nanoparticles at room temperature under visible light. In this case, surface plasmons excited in the Au nanoparticles decay into hot electrons which are transferred into a Feshbach resonance of a dihydrogen molecule on the Au nanoparticle surface, causing H_2_ dissociation [26]. Thus, the technological potential in the use of hot electron-based systems is promising. However, there are many challenges in achieving an efficient extraction of energetic electrons and holes. The main limiting factors are the short lifetimes of excited carriers in a metal, the slow transfer of momentum from a nanoparticle to plasmonic electrons and the reflection of carriers at interfaces. Previously, it was reported that embedding plasmonic structures into the semiconductor results in substantial increases in hot electron emission [37]. Also recently, we have theoretically shown that the efficiency of generation and injection of plasmonic carriers can be increased by choosing appropriate sizes, geometries and excitation frequencies [21,40].

In this work, for the first time, we offer a combination of electrophoretic and sintering approaches to produce new Au-TiO_2_ nanocomposites with high efficiency of hot electron injection. We also provide theoretical modelling of the electron generation mechanisms and for the first time calculate the contribution of hot charge carriers. We demonstrate that gold nanoparticles can be deposited into porous TiO_2_ films using an electrophoretic approach, whereby particles migrate into the TiO_2_ mesoporous electrode under the influence of an electric field. Crucially, in this paper, we show that a thermal treatment of the electrodes allows us to control both the optical properties of the electrodes and the efficiency of the photocurrent derived from hot electrons. Our thermal treatment approach opens up opportunities for increasing the photoconversion efficiency of pre-existing devices, based upon plasmonic photocurrent generators. It may also find promising applications in photosensing and devices for optical detection based upon plasmonic absorbers.

## Methods

### Material preparation

4-(Dimethylamino)pyridine (DMAP)-stabilised gold nanoparticles synthesised in water were transferred to the organic phase using a previously published method [41]. Water-soluble gold nanoparticles were synthesised by dissolving hydrogen tetrachloroaurate(III)trihydrate (0.150 g) in water (12 ml). To this solution, a solution of DMAP (0.250 g) in chloroform (12 ml) was added and stirred vigorously. The solution turned bright orange after a period of approximately 20 min, indicating the phase transfer and complexation of the DMAP ligand to the gold complex. After a further 2 h of stirring, the phases were separated and the aqueous phase was reduced with 700 μl of a solution of sodium borohydride (0.1 g) in water (10 ml). The resulting ruby red solution was stirred for a further 1 h before the phase transfer. The phase transfer of the gold nanoparticles to chloroform (CHCl_3_) was carried out by first diluting the stock gold nanoparticle solution (2 ml of stock solution in 10 ml of H_2_O). The diluted solution was then added to CHCl_3_ (10 ml) containing dodecanethiol (DDT) (830 μl). The two phases were stirred vigorously for 2 h allowing for the particles to be transferred to the CHCl_3_ layer. Once the particles had been transferred to CHCl_3_, they were further diluted with CHCl_3_ before being used for the electrophoretic deposition of the nanoparticles into TiO_2_ films and for high-resolution transmission electron microscopy (HRTEM) analysis.

Nanoparticulate TiO_2_ films were fabricated using the screen printing method onto FTO glass substrates (Sigma, Cream Ridge, NJ, USA, 2.3 mm, 13 Ω^−1^). The glass was thoroughly cleaned in a detergent solution followed by washing with isopropanol prior to deposition. In order to facilitate a good adhesion of the TiO_2_ nanoparticle layer, an initial bulk layer of TiO_2_ was deposited through drop casting an aqueous solution of TiCl_4_ (40 mM) onto heated glass substrates. The nanoparticulate TiO_2_ layer was deposited using the Dyesol 90-NRT commercial TiO_2_ paste from Dyesol Ltd., Davis CA, USA, using a 90-T polyester mesh. A single deposition allowed for the formation of a ~3-μm layer of TiO_2_ onto the FTO substrates. The dimensions of the electrodes measured 1 cm × 3 cm. After deposition, the electrodes were treated to a sintering profile of 125°C for 5 min, 350°C for 15 min, 450°C for 15 min and finally 500°C for 15 min. A ramp rate of 8°C min^−1^ was used for all steps. Once cooled, the electrodes were used for electrophoretic deposition (EPD).

EPD was carried out by submerging a TiO_2_ electrode and a blank FTO electrode into a solution of gold nanoparticles in CHCl_3_ (30 ml; 37.9 mM). Both electrodes were separated by an insulating spacer (4 mm) which ensured the distance between both electrodes was constant. A DC voltage of 250 V was applied across the electrodes for 15 min. Upon removal of the electrodes from the solution, the electrodes were rinsed with propanol and dried before further use.

The [Co(II/III)bpy_3_](PF_6_)_2/3_ redox couple was synthesised according to the procedure outlined in the reference [34].

### Material characterisation

HRTEM images were captured using a FEI Titan-High-resolution electron microscope (FEI, Hillsboro, OR, USA) operated with a beam voltage of 300 KeV. SEM images and corresponding EDX spectra were captured through analysing the side profile of the composite electrodes on a Zeiss ultra plus-scanning electron microscope (Carl Zeiss, Inc., Oberkochen, Germany) with a beam voltage of 1.5KeV.UV–vis spectra were recorded using Agilent Technologies, Cary 60 UV–vis spectrometer (Agilent Technologies, Inc., Santa Clara, CA, USA).

Photophysical measurements (photoaction response, PEC analysis, IV) were obtained using a three-electrode electrochemical cell with an Au-TiO_2_ composite WE, FTO CE, and a saturated calomel reference electrode (KCl). The electrolyte used was 0.05 M NaOH in water. Tests were carried out in a specially designed quartz cuvette (innovative lab supply), which allowed for the electrodes (1 cm × 3 cm) to be fully immersed in the electrolyte. Data was recorded with an Autolab(III) potentiostat and the Nova 1.10 software package. CVs were recorded in a standard three-electrode electrochemical cell utilising a gold working electrode (3 mm^2^), a Pt wire counter electrode and a saturated calomel reference electrode (KCl).

Incident photon-to-conversion efficiency (IPCE) data was recorded using a 150-W xenon discharge lamp. The output beam was passed through a monochromator followed by an optical chopper. The monochromatic light was chopped at a frequency of 90 Hz and monitored using an oriel instruments spectrograph (model 77400) which was calibrated using a series of laser cutoff filters (THOR Labs). The power of the frequency dependent light was calculated using a Si photodiode (Newport 818-UV-L; Newport Corporation, Irvine, CA) which outputs the frequency-dependent signal to a lock-in-amplifier. IPCE data were recorded for both the unmodified TiO_2_ electrode and the Au-TiO_2_ photoelectrode. Cells were fabricated in a sandwich configuration with a 25-μm Surlyn spacer, a Pt CE prepared via deposition of H_2_PtCl_6_ (aq.) and a [Co(II/III)bpy_3_](PF_6_)_2/3_ redox mediator.

The electrolyte composition for IPCE experiments was as follows, 0.22 M [Co(II)bpy_3_](PF_6_)_2_, 0.03 M [Co(III)bpy_3_](PF_6_)_3_, 0.1 M LiClO_4_ and 0.5 M tert-butyl pyridine in acetonitrile. IPCE values were calculated using the following expression:

$$ \mathrm{IPCE}\left(\%\right)=\frac{I_{\mathrm{sc}}(A)\;}{W\;(W)}\cdot \frac{1240}{\lambda (nm)}\cdot 100 $$

## Results and discussion

### Preparation and characterisation of Au-TiO_2_ nanocomposites

Gold nanoparticles have been initially synthesised in water and then transferred to the organic phase using a previously published method [41]. Transfer to the organic phase was necessary in order to avoid water splitting under the applied DC field, during the following EPD. CHCl_3_ acted as an ideal solvent for the phase transfer as its polar nature allowed for good ‘wettability’ and interaction with the TiO_2_ substrate. Analysis of the TEM images showed that the synthesised gold nanoparticles had an average size of 5.1 nm (see Additional file 1).

Nanoparticulate TiO_2_ films were produced by the screen-printing method onto FTO glass substrates and used for EPD. EPD is a versatile approach for depositing a wide range of materials from quantum dots [33,42], nanoparticles [43,44], polymers [45] and carbon nanomaterials [46-48]. EPD allows for charged colloidal particles, suspended in solution, to migrate under the influence of an electric field and to be deposited onto a conductive electrode of opposite charge. This approach is an extremely versatile method for the deposition of particles into a porous TiO_2_ network, allowing a range of nanoparticle deposition concentrations to be achieved, which also show an even distribution across the depth of the film. By keeping the voltage and the time of the depositions constant, it is possible to vary the concentration of gold nanoparticles in the TiO_2_ film by simply changing the concentration of gold nanoparticles in the deposition solution.

**Figure 1:**
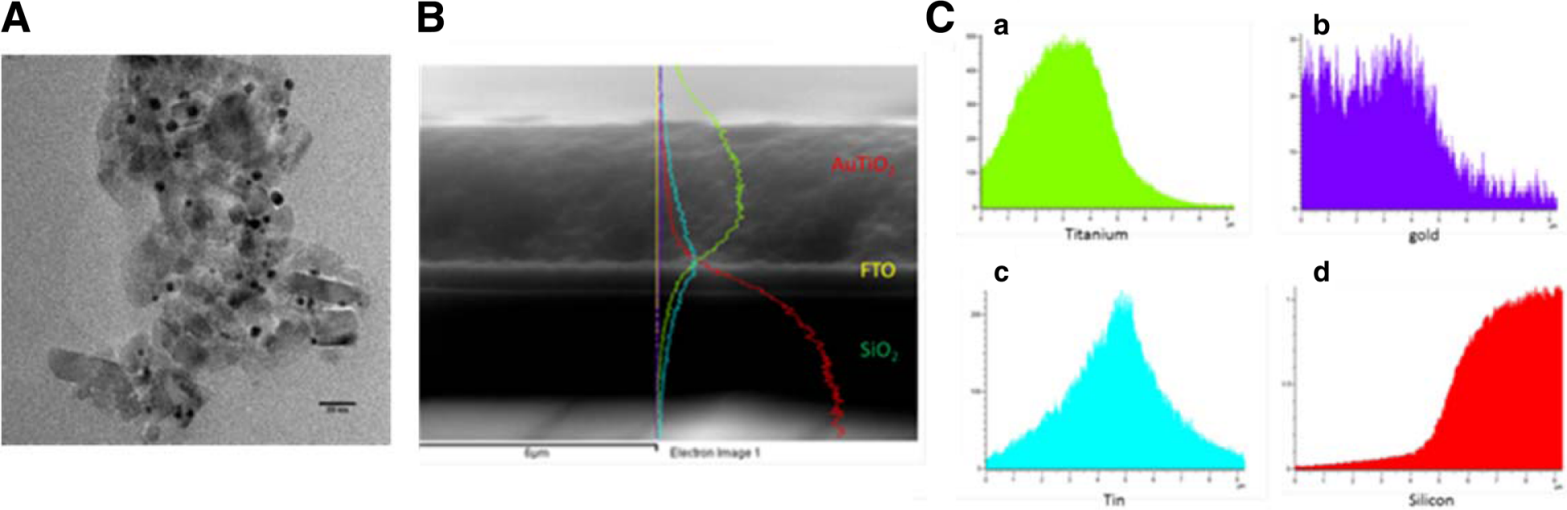
**TEM image of gold nanoparticles and EDX line mapping. A** TEM image of gold nanoparticles adhered to the surface of TiO_2_ after EPD. **B** EDX line mapping recorded for a- TiO_2_, b- gold, c- tin and d- silicon. **C** EDX spectra of a- Ti, b- gold, c- tin and d- silicon.

**Figure 2:**
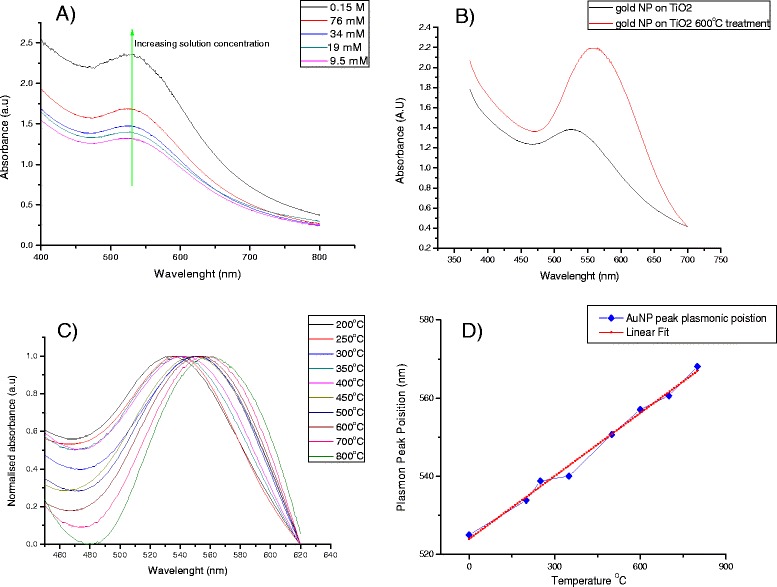
**UV–vis spectra and linear relationship between increasing sintering temperature and plasmonic peak position. A** UV–vis spectra of gold nanoparticles electrophoretically deposited into TiO_2_ films from a range of solution concentrations at 250 V for 15 min. **B** UV–vis spectra showing the shift in the plasmon peak position and the increase in plasmonic intensity after heat treatment of the Au-TiO_2_ composite films at 600°C. **C** UV–vis spectra showing the shift in the plasmon peak position with the heat treatment. **D** The corresponding linear relationship between increasing sintering temperature and plasmonic peak position.

In our work, the EPD was carried out by submerging a TiO_2_ electrode and a blank FTO electrode into a solution of gold nanoparticles. A DC voltage of 250 V was applied across the electrodes for 15 min. It was observed that gold nanoparticles were favourably deposited into the TiO_2_ film (see TEM in Figure 1) rather than on the surface of the FTO coating, which would agree with findings by Kamat et al. who have also observed this trend [49]. UV–vis spectra (Figure 2A and Additional file 1) of gold nanoparticles electrophoretically deposited into TiO_2_ films have shown increasing plasmonic intensity with the growing concentration of nanoparticles in the deposition solution. As expected, the deposition of gold nanoparticles resulted in a very large increase in the optical absorption of the TiO_2_ films when studied with UV–vis spectroscopy and visually (see Additional file 1). The presence of a large plasmon band was also observed in the UV–vis spectra, indicative of the presence of gold nanoparticles in the TiO_2_ films. The plasmon peak position of the gold nanoparticles embedded in TiO_2_ closely matched that of the plasmon position for the gold particles in the liquid phase, confirming that after the EPD, the particles are still in the nanoparticulate form and have not coalesced into a bulk gold film.

Scanning electron microscopy (SEM) of the composite films (see Additional file 1) clearly demonstrated that the EPD allowed for individual gold nanoparticles to be deposited into the TiO_2_. Some clustering of the nanoparticles was observed in the SEM images, in particular on top of the film; however, throughout the film, the particles seemed to be largely deposited individually.

Analysis of the resulting electrodes was carried with the use of energy dispersive X-ray spectroscopy (EDX) allowing the elemental composition of the composite films to be determined. The EDX spectra (Figure 1) were recorded by mapping the elemental composition of the electrode with respect to depth, recording the composition from several microns above the film to the glass substrate below. From EDX data, it is evident (Figue 1) that the gold nanoparticles are distributed evenly throughout the TiO_2_, which infers that EPD of the gold nanoparticles allowed for the particles to migrate through the pores of the TiO_2_ under the applied field. This is highly advantageous for processes such as plasmonic charge transfer as increasing the loading of nanoparticles throughout the film should allow for a more efficient charge separation to occur.

### Thermal treatment of Au-TiO_2_ composite electrodes

In order to increase the short circuit current of these electrodes under illumination, we introduced a thermal treatment of the electrodes after deposition of the nanoparticles. The thermal treatment served as a mechanism for removal of the insulating DDT ligands used to stabilise the particles in solution and allowed for gold nanoparticles to come into closer contact with the TiO_2_ nanoparticles, therefore enabling a more efficient charge injection. It was observed that heat treatment of the films results in both a significant increase in the optical absorption of the films and also increases the plasmonic intensity (Figure 2B). The plasmonic band shifts significantly to the red (524 to 562 nm after 600°C treatment), suggesting that the particles undergo a thermal ripening process while in the TiO_2_ films, as the gold nanoparticles grow and obtain a narrower size distribution [50,51]. This is also indicated by the full-width half-maximum (FWHM) values obtained for the plasmonic peaks. The FWHM value decreases from 201 to 108 nm after the heat treatment at 600°C. We expect that Au NPs which are in close proximity to each other may fuse together through a necking process at elevated temperatures, leading to the observed red shift in the absorption spectrum. The heat treatment also serves as a versatile method of finely tuning the optical properties of the electrodes. As the temperature is increased, the gold nanoparticles grow and shift further to the red region of the spectrum. We observed that it is possible to shift the plasmonic peak position from 525 to 580 nm through heating the films for 1 h, at varying temperatures. We have found that the plasmonic peak position shifts linearly with increasing temperature (Figure 2C, D); therefore, this can serve as a highly selective method for accurately tuning the optical absorption of the electrodes after the deposition has occurred. We attribute the linear correlation between plasmonic peak position and calcination temperature to the effect of nanoparticle growth within the colloidal TiO_2_ film when heated at elevated temperatures. This Au nanoparticle growth was followed and confirmed by UV–vis spectroscopy.

In addition to the removal of the insulating ligands surrounding the particles, the thermal treatment partially fuses the gold and TiO_2_ particles, resulting in the partial embedding of gold nanoparticles into the titanium dioxide structure. The fusion of the particles is expected to lead to an enhanced injection of hot electrons when illuminated at the plasmonic frequency. This effect is partially responsible for the increased plasmonic current which is observed.

### Photo-electrochemical performance tests

Photo-electrochemical (PEC) performance tests were carried out on the electrodes in order to examine the effect of the thermal treatment. The PEC tests were carried out in a three-electrode electrochemical cell utilising an Au-TiO_2_ working electrode, FTO counter electrode and a saturated calomel (KCl) reference electrode. The cells were tested under visible light (≥425 nm) illumination; the light source was chopped using an optical chopper operating at a frequency of 14 Hz.

**Figure 3:**
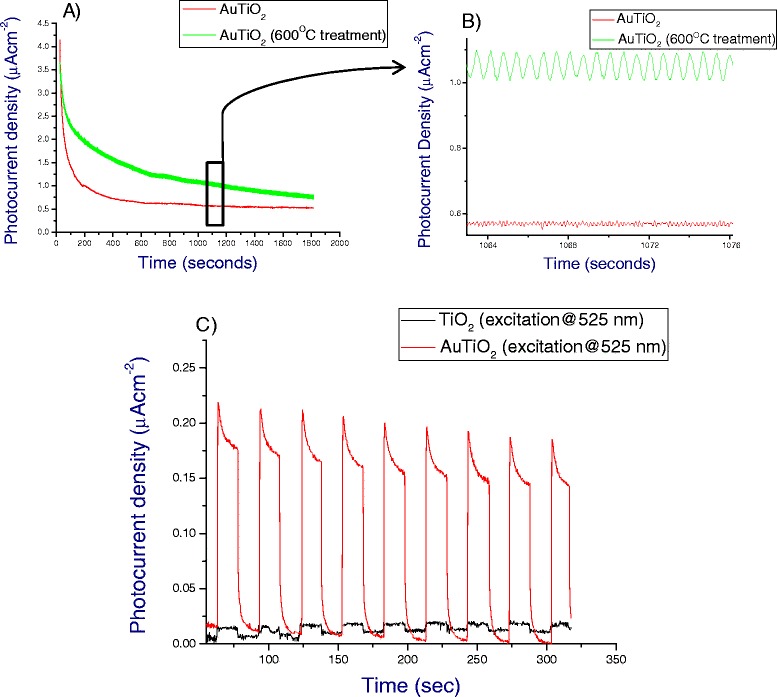
**PEC analysis. A**. Photoelectrochemical performance of heat-treated (600°C) Au-TiO_2_ film (green) and non-treated Au-TiO_2_ film (red) under visible light illumination (≥425 nm, 0.45 V vs. SCE) with a chopping frequency of 14 Hz. **B**. Highlighted region of PEC analysis. **C**. Photoaction response obtained for Au-TiO_2_ electrode when illuminated at 525 nm.

The PEC tests revealed an extremely stable and reproducible on/off switching response to the chopped light. We have observed this stable switching response at chopping frequencies greater than 100 Hz, which is indicative of a stable and fast injection response from gold to TiO_2_. PEC tests observed below (Figue 3A) were carried out at 14 Hz for clarity. The photocurrent response for the electrodes can be calculated from the difference in photocurrent observed between the on and off states (Figue 3B), whereby the on/off response is regulated by the optical chopper. The PEC analysis (Figure 3A) clearly shows an increase in the photocurrent observed for the heat-treated electrode; it can also be seen from the PEC data that the heat-treated films produce a more regular and sharper on/off switching response which would indicate the formation of a higher quality junction between the gold and TiO_2_.

It can be observed that there is also a significant increase in the magnitude of the overall current value for heat-treated electrode. The current from such a device can be attributed to a) photocurrent, b) electrolyte charge transfer properties, which depend largely on the viscosity, temperature and concentration of the solution and c) recombination. The change in photocurrent can be observed from the on/off switching states, and it is reasoned that recombination will actually be favoured for the heat-treated electrode as a photo-excited electron will not have to cross the insulating ligand barrier in order to interact with a positively charged hole. Hence, we attribute this increase in the magnitude of the overall current response to the change in the interfacial boundary layer between the gold nanoparticles and the electrolyte. If the electrodes undergo no heat treatment, then the stabilising ligands will still sit on the particle surface and limit the interaction of the particle with the electrolyte. In this work, the electrolytes employed were polar in nature (H_2_O and CHCl_3_ solvent-based systems), considering that the stabilising ligand (DDT) is a long fatty chain containing 12 carbon atoms; it can be assumed that the interaction between the particles surface and the electrolyte will be hampered by the presence of the ligands. The electrode/electrolyte boundary layer will be expected to grow over time and a current decrease will be observed. Removal of the ligands through the thermal treatment allows for a greater interaction between the particle surface and the electrolyte. The boundary layer thickness in this case is expected to decrease and for current values to increase.

As a mechanism of evaluating the photocurrent response for the electrodes, we tested the photoaction response of the electrodes under illumination at 525 nm. The photoaction response clearly demonstrated that when electrodes are illuminated at or close to the plasmonic frequency of the particles, a large photocurrent is generated comparing to the unmodified TiO_2_ (Figure 3C). A maximum photocurrent of 0.20 μA cm^−2^ is observed for the heat-treated Au-TiO_2_ electrode which is a significant increase from the 0.01 μA cm^−2^ observed for TiO_2_. This large increase in photocurrent is attributed to the generation and injection of hot plasmonic electrons from the gold nanoparticles into the TiO_2_. It can also be observed that at the plasmonic wavelength, the sintered films perform significantly better than the untreated films, owing primarily to the removal of organic ligand and the creation of a higher quality junction between gold and TiO_2_ (see Additional file 1: Figure S8).

Analysis of the photoaction response for the Au-TiO_2_ (600°C) and the uncalcinated AuTiO_2_ electrode shows an initial sharp photocurrent spike (*J*_I_), followed by a noticeable decrease in the photocurrent. After this decrease, the photocurrent reaches a steady-state value (*J*_ss_) (see Additional file 1: Figure S11). The initial spike in the photocurrent is due to the separation of plasmonic hot electrons and holes at the Au-TiO_2_ interface. Hot plasmonic electrons migrate through the TiO_2_ layer and are transported to the FTO back contact. The hot holes move to the surface of the Au nanoparticles and are captured by the reduced species in the electrolyte. The decrease in the photocurrent response, following *J*_I_, is a result of recombination processes. As holes reach the surface of the Au NPs, they may recombine with electrons in the conduction band of TiO_2_. This decay of photocurrent is determined by the rate of electron capture from holes trapped at nanoparticle surface states [52].

This effect has also been observed in colloidal TiO_2_ films when simulated with light [53,54]. In this work, the photoaction current is so small when illuminated at 525 nm that it is difficult to resolve these features in the photocurrent response.

In order to calculate the quantum efficiency of the hot electron injection from gold to TiO_2_, IPCE spectra was recorded. The cobalt mediator was chosen for these experiments as previous work using the iodide/tri-iodide redox system caused leaching of gold from the electrodes almost immediately and was deemed unsuitable for further use. This is most likely due to the formation of the stable of the stable gold (*I*) iodide species which causes the Au to leach from the electrode.

**Figure 4:**
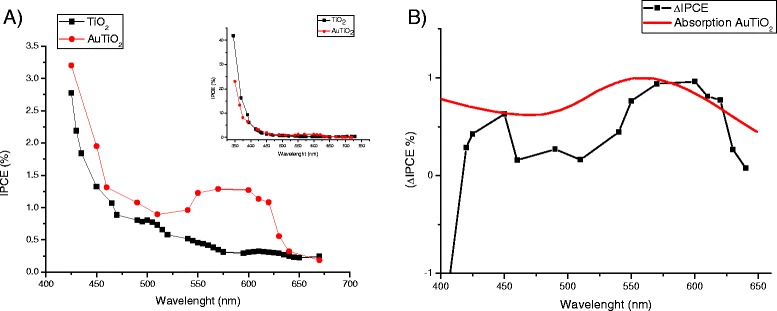
**IPCE data and ΔIPCE spectrum analysis. A.** IPCE data (recorded at 0 V) obtained for TiO_2_ and Au-TiO_2_ composite electrodes in the plasmonic domain. The inset shows the IPCE recorded from IR to UV regions of the spectrum. **B.** Experimental data for ΔIPCE and the comparison with the plasmon absorption peak (red curve).

The IPCE data (Figure 4A) have shown clear evidence for the generation of plasmonic photocurrent. Upon excitation at the plasmonic wavelength, the IPCE was observed to increase from 0.30% for unmodified TiO_2_ to 1.27% for the Au-modified system. These results are in close agreement with the photocurrent observed in the photoaction spectra (Figure 3) and also correlate closely with the UV–vis spectra obtained for the Au-TiO_2_ composite electrode, indicating that maximum photocurrent is obtained in the region of maximum plasmonic intensity.

Analysis of the ΔIPCE spectrum (Figure 4B), whereby ΔIPCE = IPCE _Au-TiO2_ − IPCE_TiO2_ reveals the contribution of only the gold nanoparticles to the overall photocurrent. The ΔIPCE shows that the gold nanoparticles affect the overall photocurrent through three distinct mechanisms. The first mechanism is the generation of hot electrons, which can be considered as a positive contributor the photocurrent production.

The second mechanism is the production of photocurrent through inter-band *d*-sp transitions within the gold nanoparticles. ΔIPCE spectra show the generation of inter-band photocurrent at 449 nm, with a quantum efficiency of 0.6% which is in exceptionally good agreement with that predicted by theory (426 nm). The photocurrent resulting for inter-band transitions can also be considered as a positive contributor to the overall photocurrent.

As the excitation wavelength is extended into the UV region, the presence of Au nanoparticles suppresses the production of current (third mechanism). IPCE values obtained at 345 nm show a decreased in photocurrent output from ~42% for TiO_2_ to ~23% for Au-TiO_2_ composite. This substantial decrease in photocurrent (44%) is attributed to the back transfer of UV-excited TiO_2_ electrons to Au nanoparticles trap states, which lie on the surface of gold. The presence of Au nanoparticles can be considered as a negative contributor to overall photocurrent when illumination is in the UV region (see Additional file 1 for ΔIPCE in UV region).

**Figure 5:**
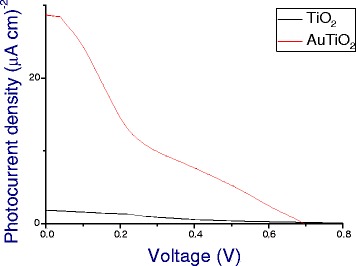
**Photocurrent dependence as a function of applied voltage for TiO**_**2**_ **and Au-TiO**_**2**_ **electrodes under excitation at 525 nm (3.0 mW).**

*I*-*V* characteristics (Figure 5) were also recorded in a three-electrode electrochemical cell under illumination at 525 nm. The dependence of the photocurrent on the applied voltage provides important information on the processes occurring in the Au-TiO_2_ electrode. The *I*-*V* data recorded shows a large increase in short circuit current for the Au-modified TiO_2_ when compared to the unmodified TiO_2_. The *J*_sc_ value increases from ~2 μA cm^−2^ to just under 30 μA cm^−2^, which is a significant increase in the photocurrent which we attribute to the plasmonic injection of hot electrons from gold nanoparticles into TiO_2_.

A theoretical discussion for all of the above processes is presented in detail in Additional file 1 and briefly outlined in the next section.

### Theoretical modelling of the contribution from the electrons and holes to the photocurrent

The previous publications [21,55] provide the theory of photo-generated electrons in plasmonic nanoparticles. It was also identified in the papers [21] and [40] that hot plasmonic holes in Au NPs are efficiently generated via the inter-band *d*-sp transitions. The rate of such inter-band transitions can be estimated from the rate of inter-band absorption in Au NPs [56]. Finally, the rate of inter-band absorption in the TiO_2_ slab can be estimated using the bulk refractive index of TiO_2_ taken from the database [57] and the calculations which are presented in Additional file 1.

**Figure 6:**
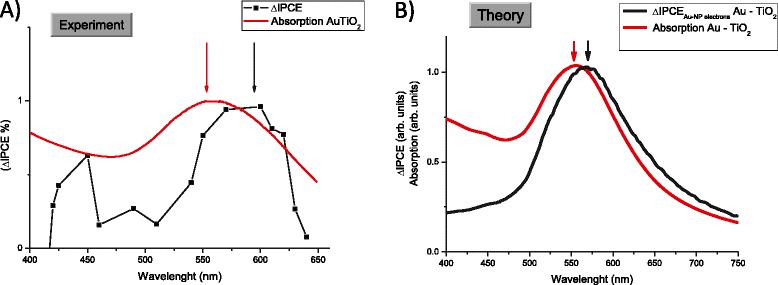
**Comparison of IPCE and absorption spectra for both experiment (A) and theory (B).** In the theoretical graph **(B)**, we show only the hot-electron contribution.

The comparison of the experimental and theoretically calculated absorption and IPCE spectra is shown in Figure 6. The plasmonic peak in the ΔIPCE in both experiment and theory is red-shifted. This effect can be explained in the following way: This peak originates from the over-barrier injection of hot plasmonic carriers. The generation rate in this case is wavelength-dependent and proportional to (see also Additional file 1):

$$ {\left|{\gamma}_{\mathrm{NP}}\left(\omega \right)\right|}^2\frac{\left(\omega -\varDelta {E}_b\right)}{\omega^4}, $$

where |*γ*_NP_(*ω*)|^2^ is the field-enhancement factor defined in Additional file 1. The above equation comes from the quantum amplitudes of optical transitions in nanocrystals. These amplitudes and the related coefficient 1/*ω*^4^ in the above equation increase with increasing the wavelength, and therefore, the position of the plasmonic maximum in the function *Rate*_NP_ (this function is given in Additional file 1) becomes red-shifted. This example shows that, in general, the photocurrent and absorption spectra are not proportional to each other. More discussions on this behaviour can be found in the publications [21,40].

**Figure 7:**
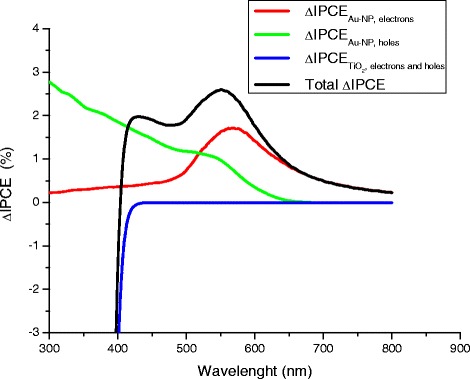
**Calculated contributions to** ***Δ*** **IPCE(** ***λ*** **).** The graph shows estimated terms *ΔIPCE*_*Au* − *NP*, *electrons*_, *ΔIPCE*_*Au* − *NP*, *holes*_, $\varDelta IPC{E}_{Ti{O}_2,\kern0.5em  electrons\kern0.5em  and\kern0.5em  holes}$, and also the calculated full spectrum ∆IPCE.

Our theory also reveals the origin of the peaks in the experimental spectrum for *Δ*IPCE(*λ*) and explains their physical nature. Figure 7 presents the following calculated features:

(1) The calculated peak at 570 nm is the plasmon resonance due to the generation of over-barrier electrons; this peak comes from the terms ∆IPCE_Au-NP,electrons_ (Additional file 1) and reflects the electric field enhancement inside the Au nanoparticles at the plasmon wave length. Correspondingly, this field-enhancement effect leads to an amplification of the hot electron injection.

(2) The structure that appears near the wavelength λ ~520 nm. This interval corresponds to the onset of the intensive inter-band generation of holes in the *d*-band of Au nanoparticles by the photons with *ω* > *ΔE*_holes_ = 2.3eV.

(3) Finally, the last structure is due to the inter-band generation of electrons and holes in the TiO_2_ film. This structure is in the interval λ <390 nm that corresponds to the inter-band absorption above the TiO_2_ bandgap for photon energies *ω* > *E*_*g*_ = 3.2eV.

Figure 7 displays the theoretical spectrum ∆IPCE and its contributions. Our calculations reproduce well the positions and signs of the contributions, but we did not attempt to calculate the magnitudes of the contributions since the dynamics and trapping of electrons and holes in the Au-TiO_2_ composite is very complex. Under light illumination used in the experiment, the system forms a steady state in which the electron population of Au NPs is constant and correspondingly, the numbers of injected electrons and holes are equal.

**Figure 8:**
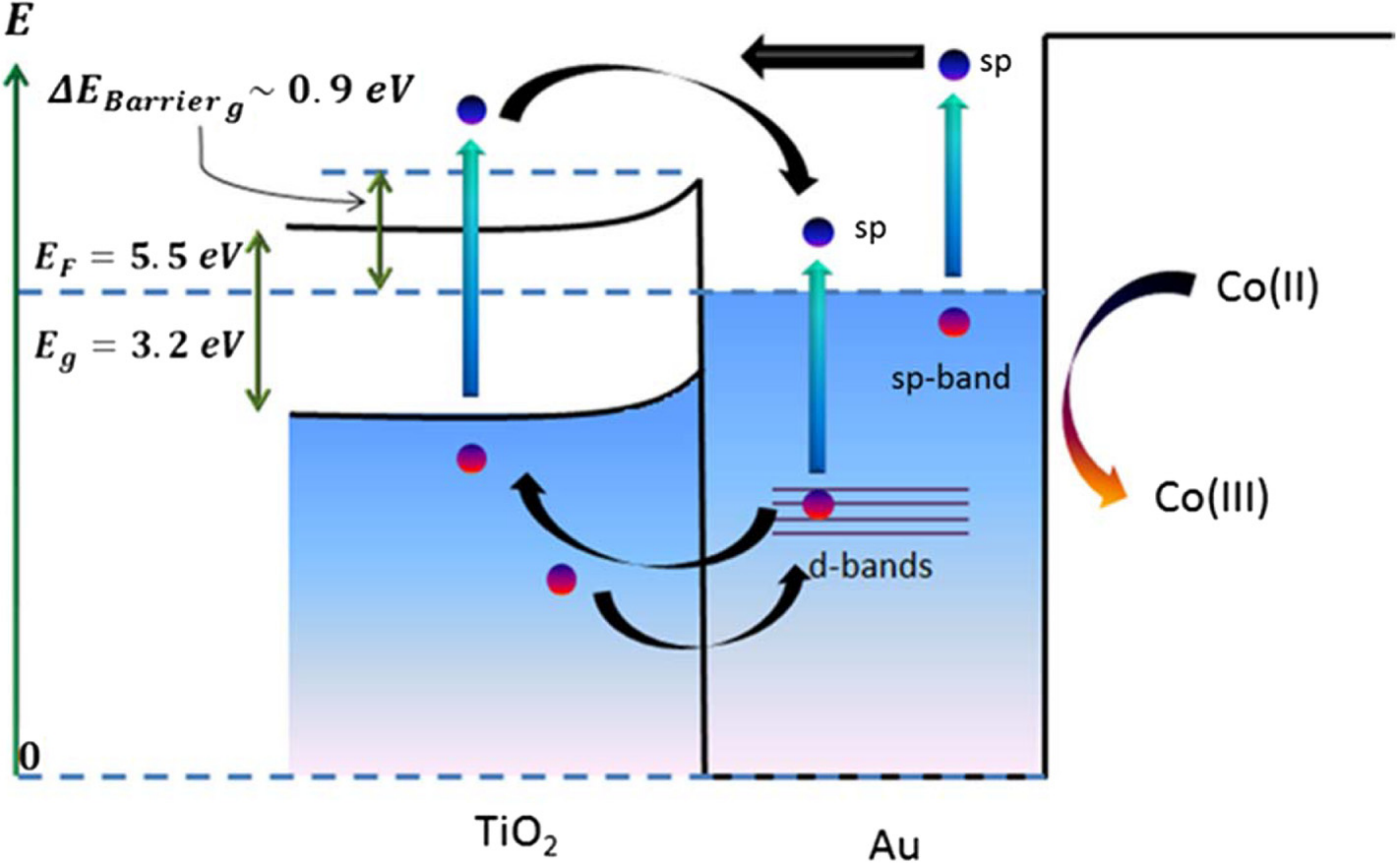
**Band diagram of the Au-TiO**_**2**_ **system and the optical and relaxation processes used in the photocurrent model.** Blue and red dots represent photo-generated hot plasmonic electrons and holes, respectively.

A schematic band diagram of the Au-TiO_2_ system and the optical and relaxation processes involved in the photocurrent model are shown in Figure 8. The hot electron–hole pair can be excited in TiO_2_ (the left-hand side) or in Au nanoparticles (the right-hand side of the Figure 8). In the case of Au nanoparticles, the hole can be excited in the sp-bands or in the *d*-band. The excitation of hole in the *d*-band is especially prominent since such holes have a large density of states. Regarding the hot electrons generated in the sp-band of Au NP, these electrons are generated from both sp- and *d*-bands (two vertical blue arrows in Figure 8). When an electron is excited from the sp-band via the intra-band transition, its energy is high and this electron can be injected into TiO_2_. When an electron is excited from the deep *d*-band, its energy is small and this electron remains trapped in the Au NP and cannot be used for the injection. The vertical red arrows depict the optical excitation processes whereas the horizontal black arrows show the transport processes such as injection from a NP, trapping in a NP and electron transfer from the Co mediator.

## Conclusions

In conclusion, we have developed a new approach for introducing gold nanoparticles into nanoporous TiO_2_ films. Most importantly, we have demonstrated that the optoelectronic characteristics of these composite electrodes can be controlled precisely with the application of a thermal treatment. In addition to the control of optical characteristics, the heat treatment serves as a tool for significantly increasing the photocurrent under illumination. We have observed plasmonic injection from Au nanoparticles to TiO_2_ with a quantum efficiency of 1.27% utilising the [Co(II/III)bpy_3_](PF_6_)_2/3_ redox mediator. We clearly demonstrated that the contribution to the photocurrent consists of 3 main components:

(i) The hot plasmonic electrons of gold nanoparticles generated due to the intra-band transitions
(ii) The hot *d*-band holes of gold nanoparticles
(iii) The electrons and holes generated via the inter-band absorptionin TiO_2_.

It is important to emphasise again that under the steady-state illumination, the numbers of electrons and holes generated by a single Au nanocrystal are the same since the charge of a nanoparticle is constant. However, the spectral features due to the injection of electrons and holes to the TiO_2_ matrix appear at different wavelengths that correspond to the Au-TiO_2_ barrier and the inter-band transitions for holes in Au. Simultaneously, the steady-state condition and the built-in electric fields inside the sample at every excitation wavelength dictate that the numbers of injected electrons and holes are the same.

The presence of gold intra-band and inter-band transitions was observed as a positive contributor to the overall photocurrent. Importantly, our theoretical calculations accurately match the results obtained from the photo-physical studies providing a detailed explanation of the processes occurring at the Au-TiO_2_ interface. Our theory also reveals the origin of the peaks in the experimental spectra for *Δ*IPCE(*λ*) and explains the physics behind the spectral features. We expect that further improvements in photocurrent output can be achieved through optimization of the photoactive layer thickness and its architecture. We also believe that these gold-TiO_2_ nanocomposites may find a range of potential energy-related applications including the use as photoanode materials for solar energy harvesting in photovoltaic cells and in new types of photocatalytic and optical sensing devices.

## Additional file

Additional file 1:
**Additional information including supporting data for this article can be found in the additional file which accompanies this article.**

## Abbreviations

*I*_sc_: short circuit current
*V*_oc_: open circuit voltage
SPR: surface plasmon resonance
WE: working electrode
FTO: fluorine-doped tin oxide
CE: counter electrode
FWHM: full-width half maximum
EDX: energy dispersive X-ray spectroscopy
EPD: electrophoretic deposition
IPCE: incident photon-to-conversion efficiency
*I*-*V*: current–voltage
