# Supplementary material for: Hot plasmonic electrons for generation of enhanced photocurrent in gold-TiO2 nanocomposites
Source: Nanoscale Res Lett. 2015 Feb 5;10:38. doi: 10.1186/s11671-014-0710-5 (PMC4385105; doi:10.1186/s11671-014-0710-5)
Supplement: Additional file 1: — Additional information including supporting data for this article can be found in the additional file which accompanies this article. [file 11671_2014_710_MOESM1_ESM.pdf]

<u>Additional Information</u>

Experimental AI

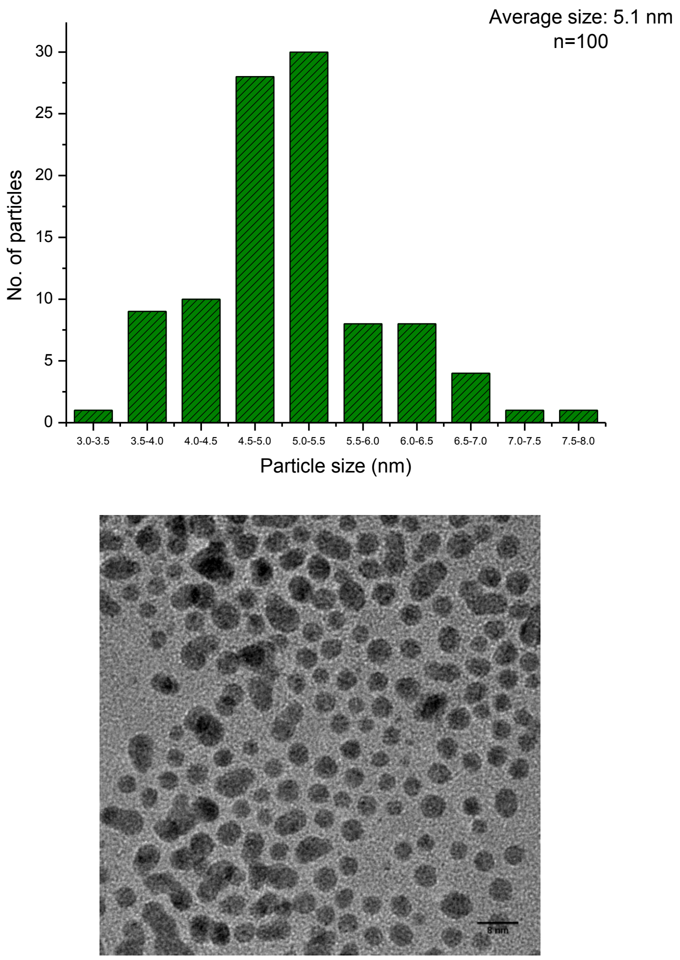

Fig. AI 1 Size distribution recorded for gold nanoparticles after phase transdfer to $CHCl_3$ and an exapmle of HRTEM image used for size distribution analysis.

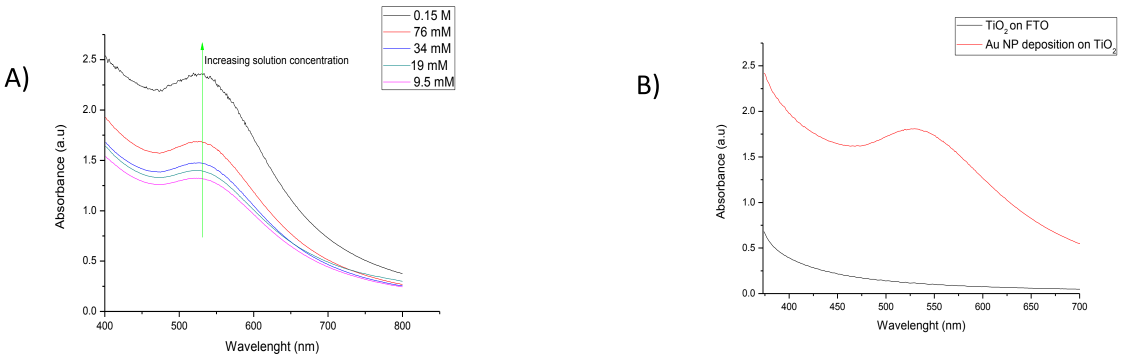

Fig. AI 2 A) Uv-vis spectra of gold nanoparticles electrophoretically deposited into TiO_2 films from a range of solution concentrations at 250V for 15 min. B) UV-vis spectra of bare TiO_2 and TiO_2 after EPD of gold nanoparticles from a solution concentration of 37.9mM

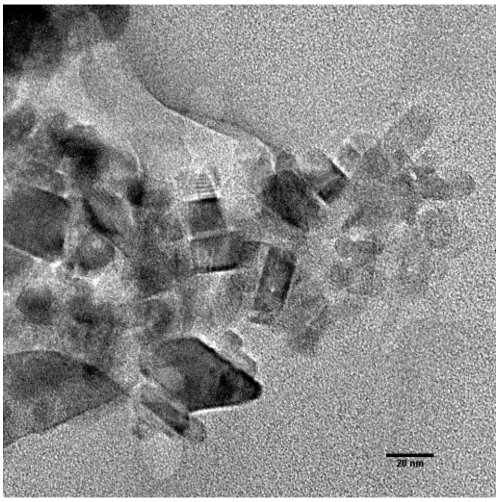

Fig AI 3 High resolution TEM image of TiO_2 nanoparticles with no gold functionalization.

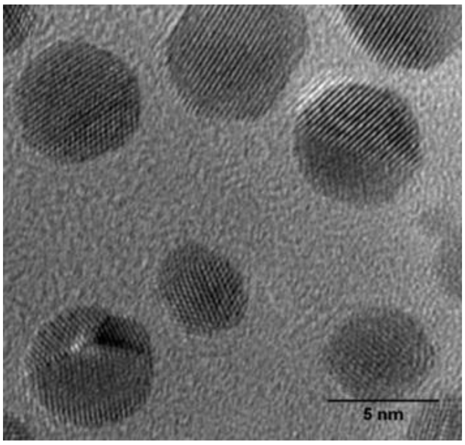

Fig. AI 4 HRTEM image of gold nanoparticles (~5nm) deposited from $CHCl_3$ onto lacy carbon grids.

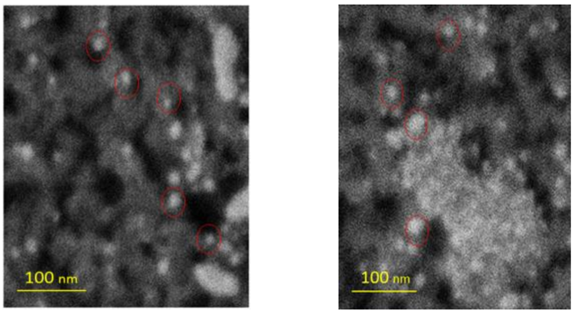

Fig. AI 5 SEM images of gold nanoparticles deposited in TiO_2 through EPD.

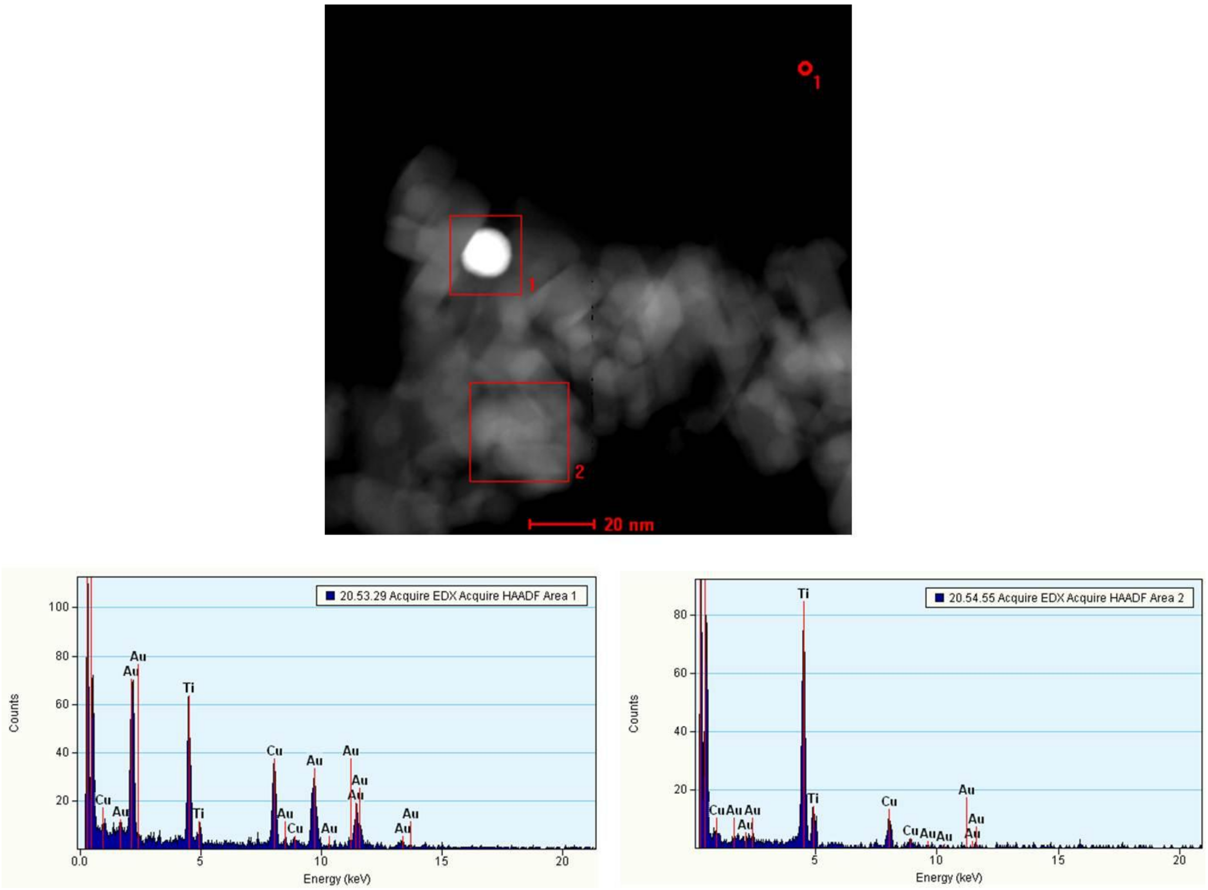

Fig. AI 6 Scanning tunnelling electron microscopy of a gold nanoparticle adhered to TiO_2 and corresponding energy dispersive X-ray spectra from highlighted regions 1 and 2.

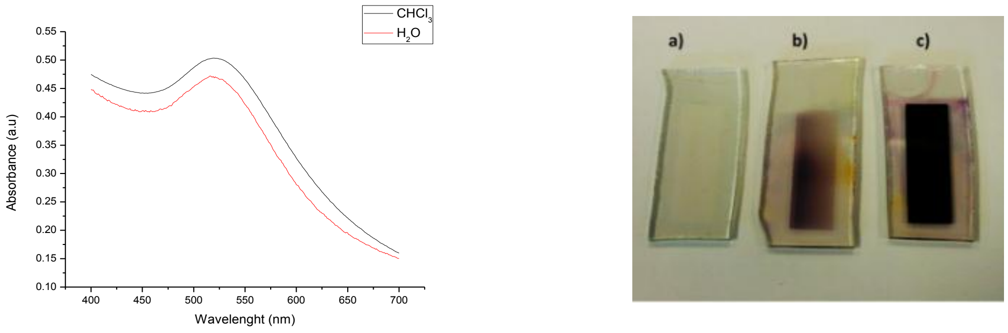

Fig. AI 7 UV-vis spectra of gold nanoparticles recorded in H_2O and after the phase transfer to $CHCl_3$. **Right:** Photographic image of a) TiO_2 b) deposition of gold nanoparticles from a solution concentration of 0.37µM c) deposition from a solution concentration of 38mM.

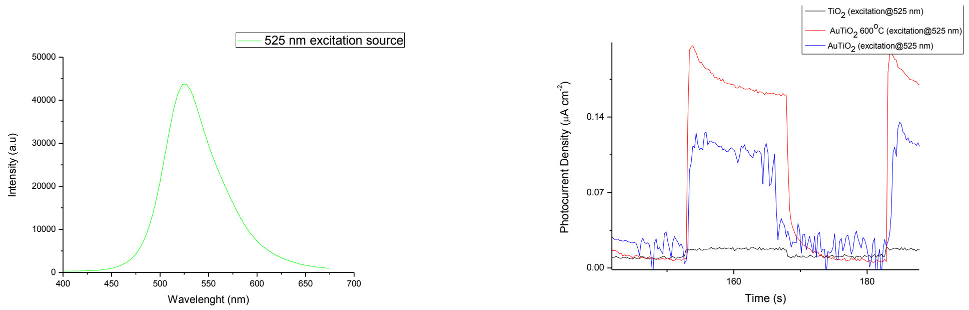

Fig. AI 8 The LED emission centered at 525 nm and the photoaction response of the untreated Au - TiO_2 composite film (blue). Photoaction response obtained for Au - TiO_2 electrode when illuminated at 525 nm (left). Photoaction results were obtained using a 3 electrode electrochemical cell with an Au - TiO_2 WE, FTO CE, and a SCRE (KCl). The electrolyte used was 0.05M NaOH in water. Tests were carried out in a specially designed quartz cuvette, which allowed for the electrodes (1cm x 3 cm) to be fully immersed in the electrolyte.

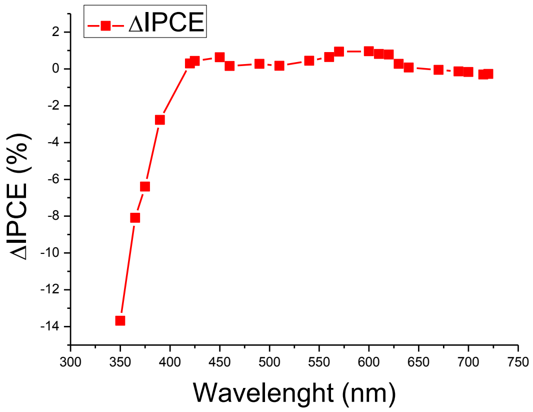

Fig. AI 9 The inset shows ΔIPCE extending into UV region.

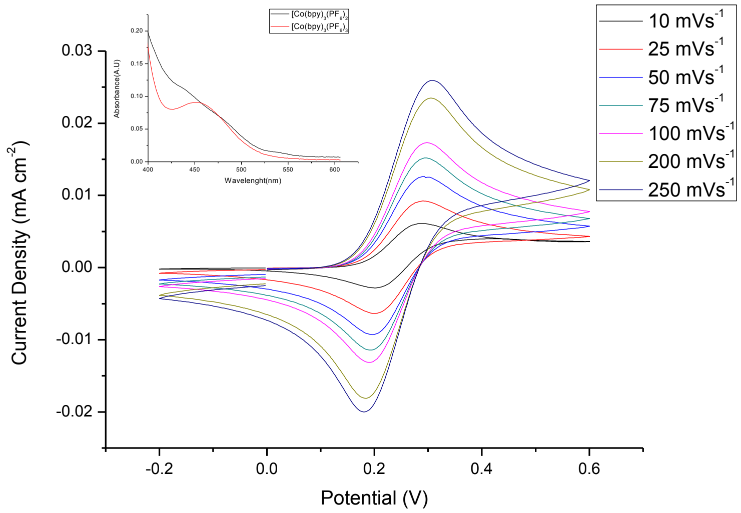

Fig. AI 10. Cyclic voltammograms recorded for the Co(II/III) redox couple in acetonitrile with increasing scan rates . CVs were recorded in a standard 3 electrode electrochemical cell utilizing a gold working electrode (3mm^2) a Pt wire counter electrode and a saturated calomel reference electrode (KCl). The inset shows UV-Vis absorption spectra recorded for both $[Co(II)bpy_3](PF_6)_2$ and $[Co(III)bpy_3](PF_6)_3$.

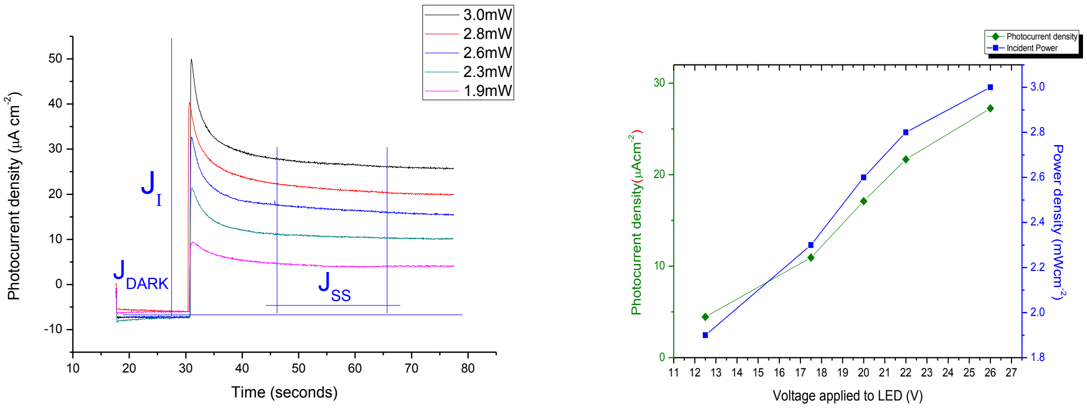

Fig. AI 11 Linear relationship between input power (mWcm^{-2}) on plasmonic photocurrent (μA; @50 s) generation.

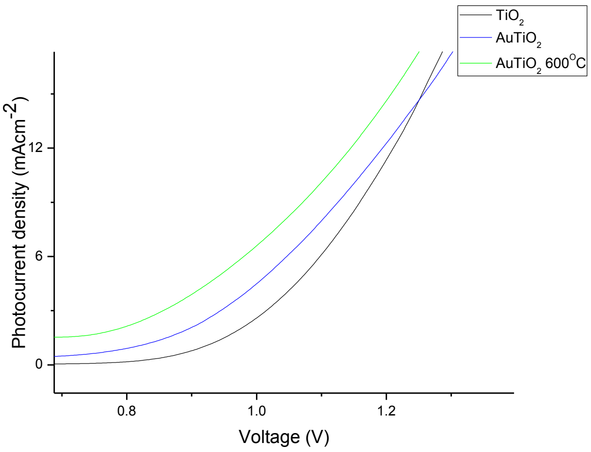

Fig. AI 12 Linear sweep voltammograms of the electrodes under visible illumination (≥ 425 nm)

Theory and Modelling

General structure of the photo-current in the samples

To understand the structure of the spectrum of $\Delta IPCE(\lambda)$ shown in Figure 4, we now present the photo-injected current in a sample as a sum of three terms since the photocurrent has its origin in three processes:

$$I_{tot} = I_{Au-NP,\, electrons} + I_{Au-NP,\, holes} + I_{TiO_2,\, electrons\ and\ holes}\,, \qquad \text{(S1)}$$

where the first two terms describe the photocurrents coming from the injection of electrons and holes from the plasmonic Au NPs, respectively, and the last term is the bulk photocurrent in the TiO_2 matrix. Figure 8 in the main text illustrates the corresponding processes. The change in the IPCE due to the presence of Au NPs is defined as

$$\Delta IPCE = IPCE_{TiO_2-Au} - IPCE_{TiO_2} = \frac{I_{tot,TiO_2-Au} - I_{tot,TiO_2}}{I_{photon}} \cdot 100\%,$$

where $I_{photon} = I_0 \cdot A / \hbar\omega$ is the rate of incident photons hitting the sample of the area A and I_0 is the incident flux. Using Eq. S1, we can now split the change of IPCE into three contributions,

$$\Delta IPCE = \Delta IPCE_{Au-NP,\,electrons} + \Delta IPCE_{Au-NP,\,holes} + \Delta IPCE_{TiO_2,\,electrons\ and\ holes}\,. \quad \text{(S2)}$$

We now look at the individual contributions.

Theoretical modelling of the contribution from the hot plasmonic electrons of Au NPs

The rate of generation of electrons above the Au-TiO_2 barrier energy, $\Delta E_{Barrier,\,e} = 0.9eV$, is given by the following integral [S1,S2]:

$$Rate_{NP} = Rate_{\text{excited electrons},\,\varepsilon > E_F + 0.9eV}(\omega) = \int_{E_F+0.9eV}^{+\infty} \delta Rate(\varepsilon)\,d\varepsilon$$

$$\delta Rate(\varepsilon) = \frac{4}{\hbar} \sum_{\alpha=(n,l,m)} \sum_{\alpha'=(n',l',m')} (f^0_{\alpha'} - f^0_{\alpha}) \left| e \cdot \varphi_{\alpha,\alpha'} \right|^2 \times$$
$$\Phi(\varepsilon - \varepsilon_\alpha) \left[\frac{\Gamma}{(\hbar\omega - \varepsilon_\alpha + \varepsilon_{\alpha'})^2 + \Gamma^2} + \frac{\Gamma}{(\hbar\omega + \varepsilon_\alpha - \varepsilon_{\alpha'})^2 + \Gamma^2} \right]$$

where $\delta Rate(\varepsilon)$ is the rate of excitation of electrons to the states with the excited energy ε; This equation is similar to the Fermi's golden rule for the transition probability. Then, we obtain the total rate of excitation above the barrier by integrating this probability. The coefficients $\varphi_{\alpha,\alpha'}$ are the matrix elements of the electric potential for the quantum states α

and α' in a spherical quantum well; $\Phi(\varepsilon-\varepsilon_\alpha)$ is a numerical delta function. Then, the total rate of generation of electrons in a sample is given by $Rate_{sample}=N_{NP}Rate_{NP}$, where N_{NP} is the number of Au NPs in a sample. Then, we assume that the corresponding contribution to IPCE is proportional to the generation rate and inversely proportional to the photon rate:

$$\Delta IPCE_{Au-NP,\,electrons}\propto\frac{Rate_{sample}}{I_{photon}}=\rho_{s,NP}\frac{Rate_{NP}}{I_0/\hbar\omega},$$

where $\rho_{s,NP}$ is the surface density of NPs in the sample that was estimated for our system to be $\rho_{s,NP}\sim\left(12nm\right)^{-2}$.

The contribution of the hot holes of Au NPs to the photocurrent

The rate of inter-band absorption is given by the dielectric function of the metal [S3]:

$$Q_{\text{inter-band}}=I_0\cdot\sigma_{\text{abs,inter-band}}=I_0\cdot\frac{1}{\sqrt{\varepsilon_0}}V_{NP}\omega\left|\frac{3\varepsilon_0}{2\varepsilon_0+\varepsilon_{metal}}\right|^2\text{Im}[\varepsilon_{\text{Au, inter-band}}],$$

where the inter-band contribution to the Au dielectric constant is given by

$$\varepsilon_{\text{Au, inter-band}}=\varepsilon_{Au,bulk}(\omega)+\frac{\omega_p^2}{\omega^2+i\omega\cdot\Gamma_D},$$

where $\varepsilon_{Au,bulk}(\omega)$ is the experimental dielectric function of gold [S4] and the second term is the Drude contribution. The dielectric function of metal, ε_{metal}, will be discussed below. The rate of generation of the holes in the d-band of Au is then calculated as $Rate_{NP,holes}=Q_{\text{inter-band}}/\hbar\omega$. The corresponding contribution to IPCE becomes

$$\Delta IPCE_{Au-NP,\,holes} \propto \rho_{s,NP} \frac{Rate_{NP,holes}}{I_0 / \hbar\omega}.$$

The contribution from the electrons and holes generated *via* inter-band absorption in TiO_2

The rate of inter-band absorption in the TiO_2 slab can be estimated as

$$Q_{TiO_2} = I_0 \cdot A \cdot (1 - e^{-2\cdot k_0 L \cdot n_2}),$$

where L , k_0 and n_2 are the film width, vacuum wave vector of light and imaginary part of the refractive index, respectively. Then, the rate of degeneration of electron and holes in the whole sample $Rate_{TiO_2,tot} = Q_{TiO_2} / \hbar\omega$. The effect of Au NPs comes from the probability to trap electrons and holes inside NPs removing such carriers from the photo-current,

$I_{TiO_2-Au} - I_{TiO_2} = P_{trap} \cdot Rate_{TiO_2,tot}$, where P_{trap} is the trapping probability. The related term in IPCE then becomes

$$\Delta IPCE_{TiO_2,\,electrons\ and\ holes} = \frac{P_{trap} Rate_{TiO_2,tot}}{I_{photon}} \cdot 100\% = P_{trap} (1 - e^{-2\cdot k_0 L \cdot n_2}) \cdot 100\%.$$

The trapping probability, P_{trap}, is an unknown parameter and depends on the dynamics and spatial distribution of photo-generated carriers. Since the absorption rate in the 3-μm TiO_2 film is very strong in the UV and starts abruptly at 391nm, our result does not depend much

on the coefficient P_{trap}. Basically the contribution $\Delta IPCE_{TiO_2,\, electrons\ and\ holes}$ looks like a negative step function at 391nm (Figure 7 for $\Delta IPCE(\lambda)$). To be definitive, we took $P_{trap} = 0.3$.

The spectrum $\Delta IPCE$

Figure 7 displays the modelled spectrum $\Delta IPCE$ and its contributions. Our calculations reproduce well the positions and signs of the contributions, but we did not attempt to calculate the magnitudes of the contributions since the dynamics and trapping of electrons and holes in the Au -TiO_2 composite are very complex. In Figure 7, we used arbitrarily weights of the three contributions to the spectrum $\Delta IPCE(\lambda)$ given by Eq. S2.

Fitting of the theoretical dielectric function to the experimental absorption

We use here the standard approach to introduce an additional broadening to the empirical dielectric function of pure gold. It is typical that the plasmon resonances of real nanostructures are broader than the ones calculated using the dielectric constant of bulk metal. This occurs due to defects in a crystal and also finite size effects. Therefore for better agreement with the experiment, we introduced an additional broadening in the local dielectric function using the standard approach [S5]:

$$\varepsilon_{metal}(\omega) = \varepsilon_{Au,bulk}(\omega) + \frac{\omega_p^2}{\omega^2 + i\omega \cdot \Gamma_D} - \frac{\omega_p^2}{\omega^2 + i\omega \cdot \Gamma_{D,broadened}},$$

where $\Gamma_D = 0.076eV$ and $\omega_p = 8.9eV$ are the Drude parameters taken from the fitting of the experimental dielectric function of gold, $\varepsilon_{Au,bulk}(\omega)$, in the long wavelength region; $\Gamma_{\mathrm{D},broadened} = 7 \cdot \Gamma_{bulk,D}$ is the new, increased broadening used to describe the present

experimental data. Additional fitting parameter in our problem is the dielectric constant of the matrix. TiO_2 is highly porous and its dielectric constant is expected to be lower than the bulk one. Indeed, from the fitting to the experimental position of the plasmon peak we got $\varepsilon_0 = 3$, whereas the bulk value for TiO_2 at 600nm is ~ 8.4. Figure SI13 below shows the result of fitting.

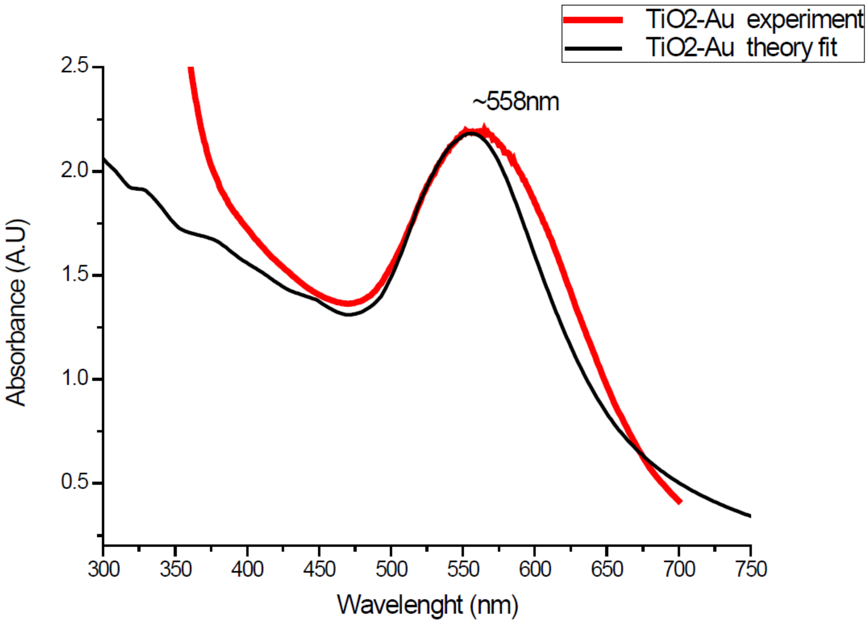

Fig. AI 13 Experimental absorption and the fitted theoretical spectrum coming from the Mie theory and the broadened dielectric function of gold.

AI references:

[A1] Govorov, A. O., Zhang, H. & Gun'ko, Y. K. Theory of Photoinjection of Hot Plasmonic Carriers from Metal Nanostructures into Semiconductors and Surface Molecules. J. Phys. Chem. C 2013, 117, 16616-16631.

[A2] Zhang, H. & Govorov, A. O. Optical Generation of Hot Plasmonic Carriers in Metal Nanocrystals: The Effects of Shape and Field Enhancement. J. Phys. Chem. C 2014, 118, 7606-7614.

[A3] Govorov, A.O., Zhang, H., Demir, V., Gun'ko, Y. K. Nano Today 2014, 9, 85–101.

[A4] Johnson, P. B., Christy, R. W. Optical Constants of the Noble Metals. Phys. Rev. B 1972, 6, 4370−4379.

[A5] Kreibig, U., Vollmer, M. Optical Properties of Metal Clusters; Springer-Verlag: Berlin, 1995.
